# Draft Genome Sequence of *Mycobacterium farcinogenes* NCTC 10955

**DOI:** 10.1128/genomeA.00523-14

**Published:** 2014-05-29

**Authors:** Olivier Croce, Catherine Robert, Didier Raoult, Michel Drancourt

**Affiliations:** Unité de Recherche sur les Maladies Infectieuses et Tropicales Émergentes, CNRS, UMR 6236, IRD 198, Faculté de Médecine, Aix-Marseille Université, Marseille, France

## Abstract

We report the draft genome sequence of *Mycobacterium farcinogenes* NCTC 10955 (=DSM 43637^T^), a nontuberculosis species responsible for bovine farcy. The strain described here is composed of 6,139,893 bp, with a G+C content of 65.73%, and contains 5,816 protein-coding genes and 76 RNA genes.

## GENOME ANNOUNCEMENT

In sub-Saharan Africa, *Mycobacterium farcinogenes*, the etiological agent of bovine farcy (a form of bovine lymphangitis), has major economic implications in some resource-limited African countries (1). It was initially described in Chad and Senegal as comprising two subspecies, *tchadense* and *senegalense* (2–4), further elevated as two species, *Mycobacterium farcinogenes* and *Mycobacterium senegalense* (5). Numerical taxonomy studies confirmed these data (6), as did the 16S-23S intergenic spacer sequence analysis which further revealed that *M. farcinogenes* and *M. senegalense* belong to the *Mycobacterium fortuitum* complex (7), a group of mycobacteria that also includes *Mycobacterium conceptionense* (8). *M. farcinogenes* has also been isolated from soil (9) and rarely implicated as a human pathogen, with one case of hip prosthesis infection, yet formal evidence is lacking for an accurate identification (10).

We performed whole-genome sequencing of *M. farcinogenes* DSM 43637^T^ (=NCTC 10955) in order to precisely define its relationship with *M. senegalense* and other closely related mycobacteria and to contribute to the development of advanced molecular tools for its detection and identification.

Genomic DNA isolated from *M. farcinogenes* strain DSM 43637^T^ was grown on MGiT Middlebrook broth at 37°C. It was then sequenced using Roche-454 technology (11). Two Roche-454 libraries were constructed: a 4.9-kb paired-end and a 1.49-kb shotgun XL+. Each library was loaded on a picotiter plate and sequenced with the Roche-GS FLX Titanium Sequencing kit XLR70. The 2 runs yielded 115.66 Mb with 287,369 passed filters and an average length of 442 bp.

Reads from 454 sequencing were assembled into contigs and scaffolds using Newbler version 2.8 (Roche-454 Life Sciences). Contigs obtained were combined together by Opera software v1.2 (12) combined to GapFiller v1.10 (13) to reduce the set. Some manual refinements using CLC Genomics v7 software (CLC bio, Aarhus, Denmark) improved the genome. Finally, the draft genome of *M. farcinogenes* was found to consist of 5 scaffolds of 63 contigs containing 6,062,162 bp and an estimated size including gaps of 6,139,893 bp. The G+C content of this genome is 65.73%.

Noncoding genes and miscellaneous features were predicted using RNAmmer (14), ARAGORN (15), Rfam (16), PFAM (17), and Infernal (18). Coding DNA sequences (CDSs) were predicted using Prodigal (19) and functional annotation was achieved using BLAST+ (20) and HMMER3 (21) against the UniProtKB database (22). The genome was shown to encode at least 76 predicted RNAs including 3 rRNAs in a single operon, 57 tRNAs, 1 transfer-messenger RNA, and 15 miscellaneous RNAs. A total of 5,816 genes yielded a coding capacity of 5,610,858 bp (coding percentage: 91.3%) and included 749 (12.87%) genes encoding putative proteins, 1,023 (17.59%) genes assigned as hypothetical proteins, and 5,766 genes matching a least one sequence in the Clusters of Orthologous Groups (COG) database (23, 24) with BLASTP default parameters.

### Nucleotide sequence accession numbers.

The *M. farcinogenes* NCTC 10955 (= DSM 43637^T^) strain genome sequence has been deposited at DDBJ/EMBL/GenBank under the accession no. HG964481 to HG964485. The whole-genome shotgun master numbers are CCAY010000001 to CCAY010000063.
